# Evaluation of an Automated Proton Planning Solution

**DOI:** 10.7759/cureus.3696

**Published:** 2018-12-06

**Authors:** Alexander R Delaney, Wilko F Verbakel, Jari Lindberg, Timo K Koponen, Ben J. Slotman, Max Dahele

**Affiliations:** 1 Medical Physics, VU University Medical Center, Amsterdam, NLD; 2 Radiation Oncology, VU University Medical Center, Amsterdam, NLD; 3 Miscellaneous, Varian Medical Systems, Helsinki, FIN

**Keywords:** impt, protons, knowledge-based planning, model-based planning, automation

## Abstract

Purpose

Intensity-modulated proton therapy (IMPT) treatments are increasing, however, treatment planning remains complex and prone to variability. RapidPlan^TM^PT (Varian Medical Systems, Palo Alto, California, USA) is a pre-clinical, proton-specific, automated knowledge-based planning solution which could reduce variability and increase efficiency. It uses a library of previous IMPT treatment plans to generate a model which can predict organ-at-risk (OAR) dose for new patients, and guide IMPT optimization. This study details and evaluates RapidPlan^TM^PT.

Methods

IMPT treatment plans for 50 head-and-neck cancer patients populated the model-library. The model was then used to create knowledge-based plans (KBPs) for 10 evaluation-patients. Model quality and accuracy were evaluated using model-provided OAR regression plots and examining the difference between predicted and achieved KBP mean dose. KBP quality was assessed through comparison with respective manual IMPT plans on the basis of boost/elective planning target volume (PTV_B_/PTV_E_) homogeneity and OAR sparing. The time to create KBPs was recorded.

Results

Model quality was good, with an average R^2^ of 0.85 between dosimetric and geometric features. The model showed high predictive accuracy with differences of <3 Gy between predicted and achieved OAR mean doses for 88/109 OARs. On average, KBPs were comparable to manual IMPT plans with differences of <0.6% in homogeneity. Only 2 of 109 OARs in KBPs had a mean dose >3 Gy more than the manual plan. On average, dose-volume histogram (DVH) predictions required 0.7 minutes while KBP optimization and dose calculation required 4.1 minutes (a ‘continue optimization’ phase, if required, took an additional 2.8 minutes, on average).

Conclusions

RapidPlan^TM^PT demonstrated efficiency and consistency and IMPT KBPs were comparable to manual plans. Because worse OAR sparing in a KBP was not always associated with geometric-outlier warnings, manual plan checks remain important. Such an automated planning solution could also assist in clinical trial quality assurance and overcome the learning curve associated with IMPT.

## Introduction

The growing interest in proton therapy is substantiated by the recent increase in the number of treatment centers globally. However, it is well documented that even established photon techniques like intensity-modulated radiation therapy (IMRT) or volumetric modulated arc therapy (VMAT) suffer from a wide variation in treatment plan quality between planners and institutes [1-2]. It is, therefore, reasonable to assume that this will also be a problem for newer, increasingly complex modalities such as intensity-modulated proton therapy (IMPT). The complexity of IMPT treatment planning is amplified by, amongst other factors, beam-angle selection, use of a range shifter/bolus and robust/non-robust optimization. As a result, determining the achievable organ-at-risk (OAR) sparing for a prospective patient is difficult, often requiring multiple optimizations. Automated treatment planning, for example using model/knowledge-based planning, could assist in overcoming such difficulties [3]. One example of a solution to address the variation observed in photon treatment planning, and to try and improve planning efficiency, is RapidPlan^TM^ (Varian Medical Systems, Palo Alto, California, USA), a knowledge-based automated planning approach which has shown promising results in multiple disease sites [4-7]. In a proof-of-principle study using a version built to accommodate the physical characteristics of photons, we were previously able to show that RapidPlan^TM^ could also be used to select patients for proton therapy [8]. We have subsequently collaborated with Varian Medical Systems in the development and evaluation of a proton-specific platform, RapidPlan^TM^PT. In this article, we highlight key features of this planning tool, including differences with the photon platform, and evaluate, for locally advanced head and neck cancer (HNC), a model based on a 50-patient IMPT library using 10 validation cases which were not included in the model.

## Materials and methods

RapidPlan^TM^PT

**Figure 1:**
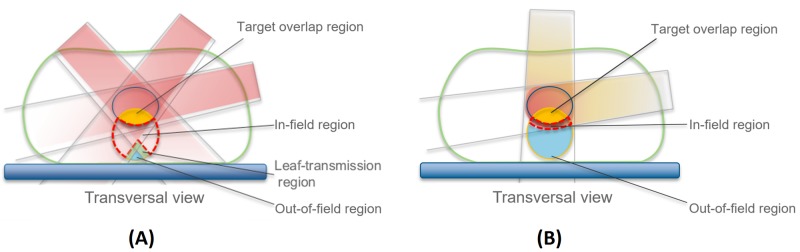
Volume partitioning in RapidPlanTM for (A) photons and (B) protons

RapidPlan^TM^ is a knowledge-based planning solution which uses the geometries and associated dosimetry contained in a library of previously created treatment plans to construct a model which can be then used to predict a range of achievable dose-volume histograms (DVHs) for the OARs of a prospective patient with pre-selected beam directions. Predictions dictate a placement of optimization objectives to automate the optimization process, resulting in a knowledge-based plan (KBP). RapidPlan^TM^ for photons has been described previously [4,6]. Model training can be divided into two phases, extraction and training. During extraction, both the dosimetry and geometry of each library plan are parameterized. In order to evaluate geometry, OARs are partitioned into the (1) out-of-field region (scattered dose), (2) leaf-transmission region (not strongly affected by optimization), (3) target overlap region (dose comparable to that of target) and, finally, (4) in-field region, which is the most heavily modulated region (Figure 1). Since (1-3) are subject to limited modulation, the average DVH for such regions is calculated along with the standard deviation. This standard deviation acts as a lower and upper boundary for the DVH-prediction. Since the in-field region is heavily modulated, more sophisticated modeling is required. The geometry is evaluated using geometry-based expected dose (GED). The GED is used to determine how the geometrical position and distribution of an OAR, relative to the target structures, affects the achievable dosimetry under the current field geometry [9]. As well as field geometries, the GED takes into account the physical characteristics and behavior of photons. The geometry of an OAR therefore comprises GED DVHs and a number of parameters such as OAR volume, OAR-planning target volume (PTV) overlap and target volume. RapidPlan^TM^ then parameterizes both the geometry and achieved DVHs using principal component analysis. Once parameterized, a regression model can be then used to predict a probable range of DVHs for each OAR of a prospective patient using their geometry. Finally, dose-volume objectives are automatically placed near the lower boundary of each OAR DVH prediction range, to guide the optimization process. The performance of the photon RapidPlan^TM^ platform has been extensively evaluated for VMAT and IMRT for optimal treatment planning of different disease sites [5,7,10-11].

RapidPlan^TM^PT, the proton-specific platform, was developed to accommodate the different physical traits of protons, through adaptation of the GED metric, which for protons is based on a simplified spread-out Bragg peak (SOBP) model. Contrary to photons, OAR partitioning in RapidPlan^TM^PT does not include a leaf-transmission region (Figure 1). Furthermore, the in-field region is the part of the OAR where the GED is above a certain threshold and with no target overlap. The out-of-field region is the region where the GED is below a certain threshold. Similar to photons the GED measures the dose received by each voxel considering target doses and the field geometry. The GED is evaluated for each field and target level. For each target level, target voxels receive the prescribed dose while other voxels receive a dose-dependent on their position along the beamline. Entry dose deposited into a voxel positioned before the target depends on the target length in the beam direction and the distance from the target. Distal fall-off determines the dose in voxels positioned after the target, being dependent on the distance of voxels from the target edge and steepness of the fall-off. A dilation operation followed by 3D Gaussian convolution is applied to the total GED distribution swelling the GED distribution and producing a smoother decay (the penumbra). If the beamline intersects the target multiple times (non-convex target) the disjoint target regions are considered as separate sub-targets when calculating the GED distribution. This reduces dose in non-target voxels between the spatially separated target regions.

IMPT treatment planning

Locally advanced HNC IMPT plans were based on a simultaneous integrated boost (SIB) technique, delivering 70/54.25 Gy to the boost/elective PTV (PTV_B_/PTV_E_) in 35 fractions. A 5-mm transition-region between PTVs was created to facilitate a gradual dose fall-off (PTV_O_). Plans typically aimed to spare the oral cavity as well as parotid glands, submandibular glands and individual swallowing muscles, although certain OARs could sometimes be excluded due to the extent of their overlap with PTVs. Laterality (ipsilateral/contralateral) of an OAR was determined by assessing which OAR was more proximal to the PTV and/or had more overlap with the PTV. The aim was to deliver 95% of the prescribed dose to 99%/98% of PTV_B_/PTV_E_ while limiting the PTV volume receiving >107% of the prescription dose. IMPT plans were created using the non-linear universal proton optimizer (NUPO, Varian Medical Systems, Palo Alto, CA, USA) v13.7.14 and proton convolution superposition (PCS, Varian Medical Systems, Palo Alto, CA, USA) algorithm v13.7.14 with a 2.5 mm dose calculation grid. Spot sigma in the air at the isocenter was 3.9 mm for 240 MeV proton beams. Spot spacing was 0.425 times the energy-dependent in-air full width half maximum (FWHM) spot size at the isocenter. Plans were made with a standard three-field, multi-field optimization (MFO) technique, with gantry angles at 35°–55°, 180° and 305°–330°. The field target for each field was the union of PTV_B_, PTV_E_ and PTV_O_, termed PTV_COMP_. A range shifter of 5.7 cm water equivalent thickness was used to allow for irradiation of proximal portions of the PTVs. Each field included typical proximal, distal and lateral target margins of 0.2 cm, 0.3 cm and 0.5 cm, respectively. Optimization was performed interactively during planning by manually adjusting optimization objectives to maintain an approximately fixed diagonal distance to DVH-lines displayed in the optimization window [12]. A subsequent “continue optimization” was used in eight of 10 cases to improve PTV dose homogeneity/coverage. Maximum point dose-objectives were used for the spinal cord, brainstem and their planning at risk volumes.

RapidPlan^TM^ model

IMPT treatment plans were created for 50 arbitrarily selected HNC patients and used to populate the model library. Model quality was evaluated using the regression, residual and DVH-plots as well as R^2^ values, which indicate the quality and variance of regression models, with 1 indicating a perfect fit between geometric and dosimetric features [11]. DVHs or points in the regression and residual plots which deviated from the bulk of the population were removed using visual analysis of the aforementioned plots in conjunction with the available statistical metrics. However, based on previous experience, extensive outlier removal was not performed [13].

Evaluation group

For an evaluation group of 10 HNC patients (not included in the model), both manual and KBP IMPT plans were created. KBPs were created using the upcoming release version of NUPO v15.7.0 and PCS v15.0.17 with a 2.5 mm dose calculation grid. Both the ­­beam arrangements and priorities of optimization objectives for KBPs were the same as in the respective manual plans. Furthermore, a “continue optimization” was performed if PTVs did not meet the aforementioned planning aims. Finally, KBPs were normalized to the same mean PTV_B_ dose as the respective manual plans.

Study endpoints

Model quality was evaluated as described above. The accuracy of RapidPlan^TM^PT predictions was determined by examining the difference between predicted and achieved mean dose for each KBP [14]. The KBPs were benchmarked against their respective manual plans on the basis of (1) the homogeneity index (HI) of PTV_B_/PTV_E_ (HI_B_/HI_E_), where (HI_B_/HI_E_) = 100x(Dose to 2% of the volume(D2%)-D98%)/D50% and PTV_B_/PTV­_E_ V95, (2) mean dose to individual OARs and combined salivary and swallowing structures (comp_sal_ and comp_swal_, respectively). The time required to create KBPs was also reported.

## Results

**Figure 2:**
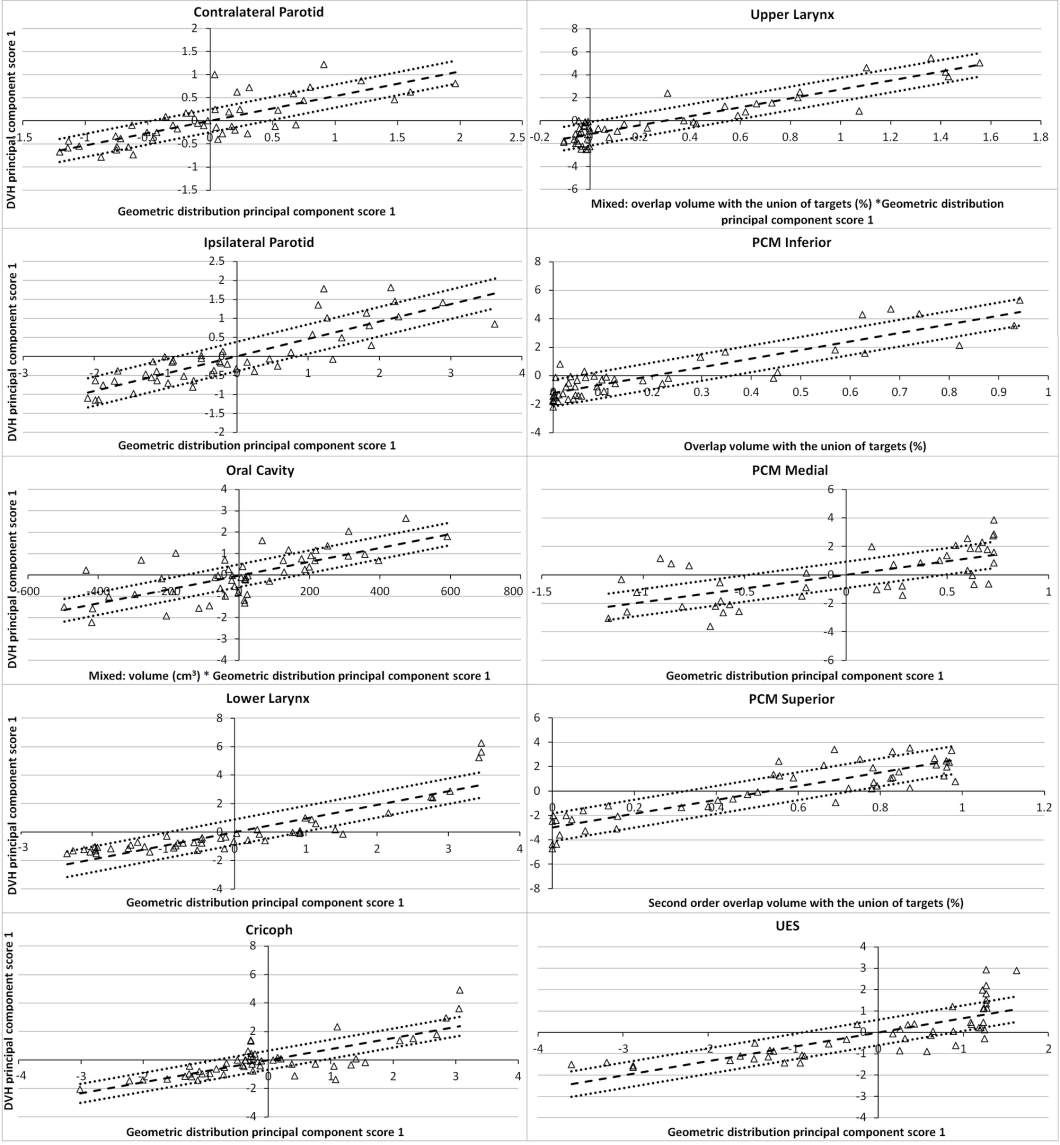
Regression plots for the parotid glands, oral cavity and swallowing structures The regression lines (dashed) and confidence intervals (dotted-line), one standard deviation, are shown for each. DVH: Dose-volume histogram

**Figure 3:**
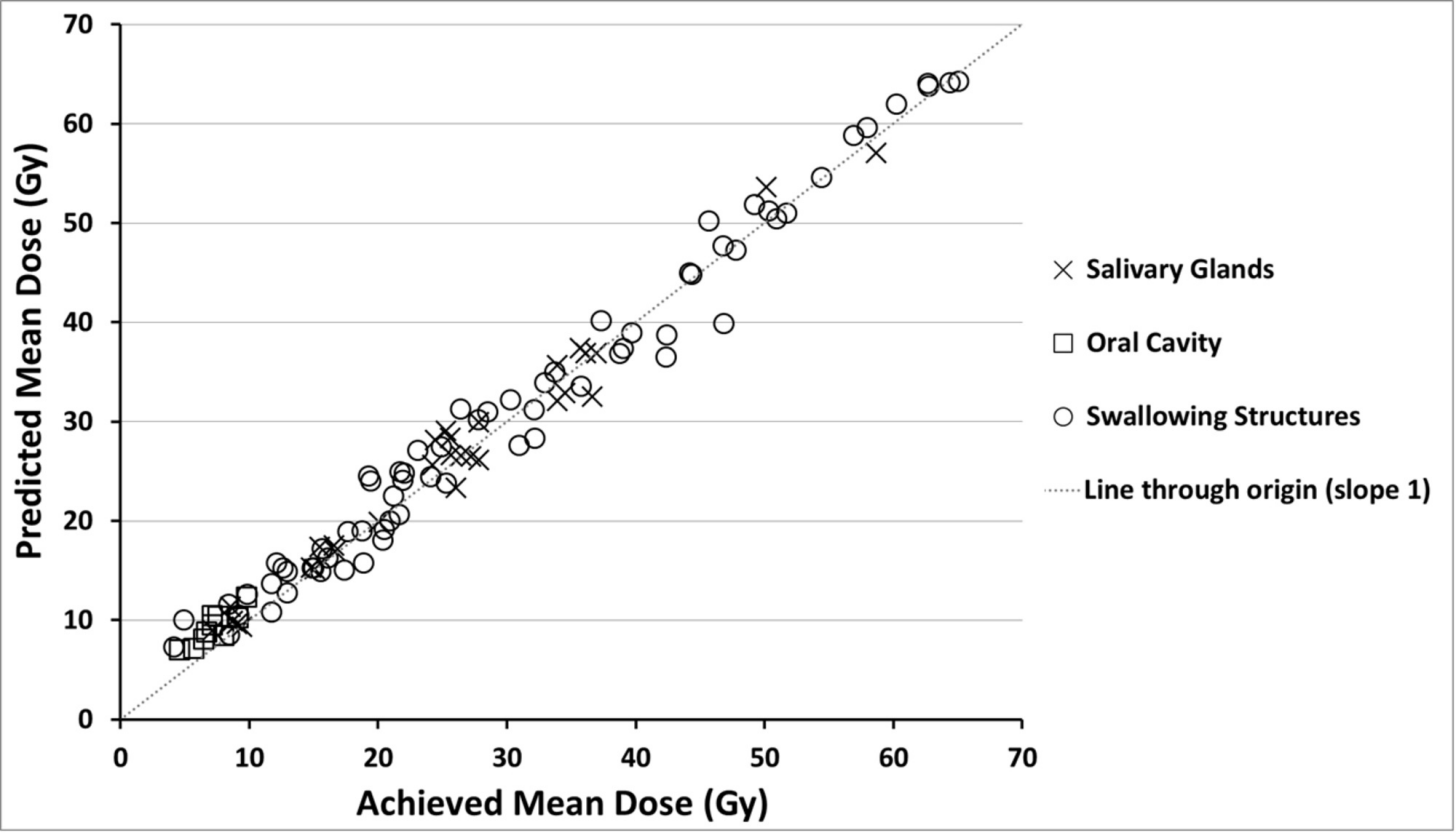
Achieved versus predicted mean dose for all 10 evaluation patient knowledge-based plans

**Figure 4:**
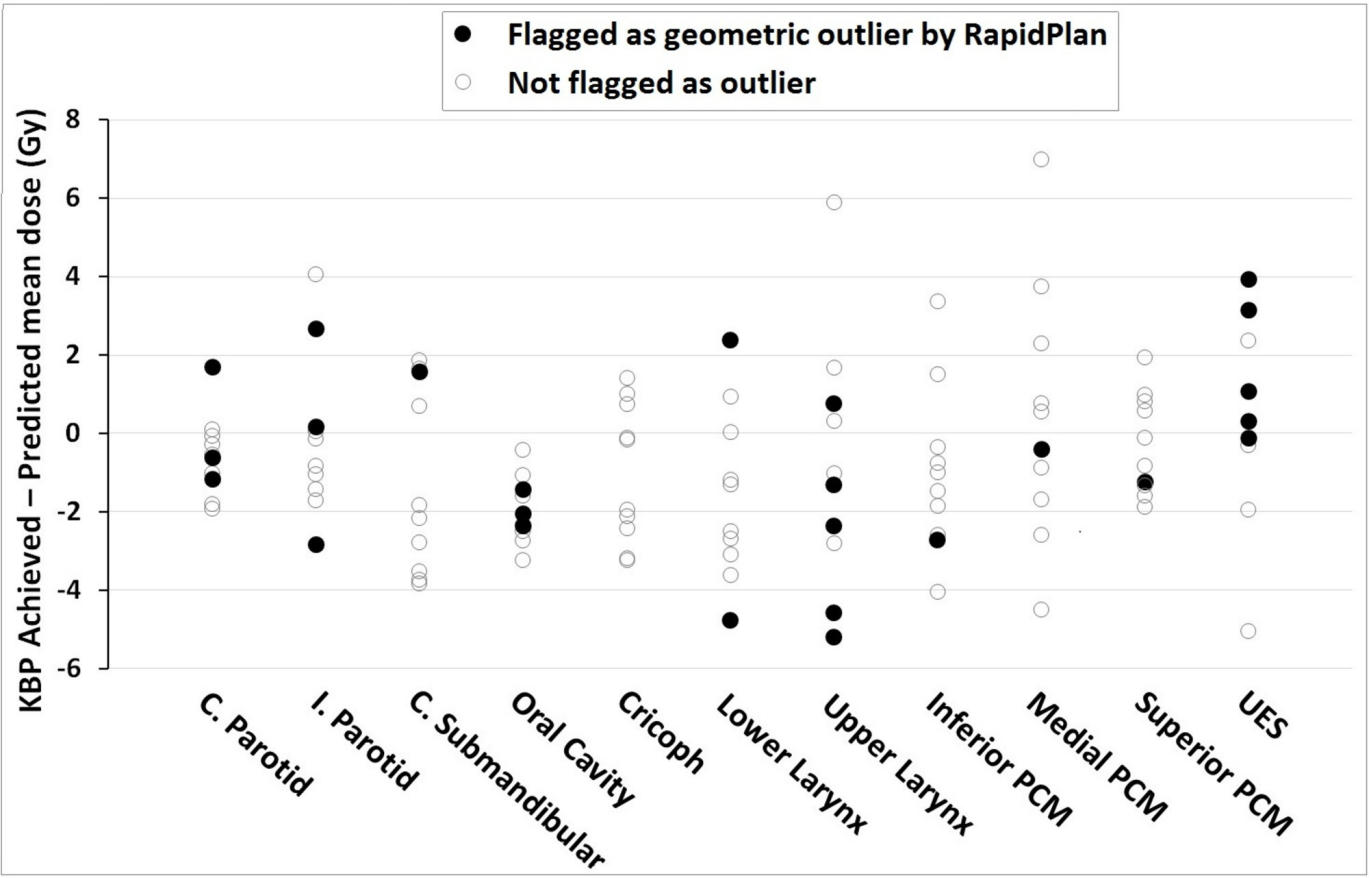
Achieved minus predicted mean dose for the OARs of all 10 evaluation KBPs C. Parotid: Contralateral parotid; I. Parotid: Ipsilateral parotid; C. Submandibular: Contralateral submandibular; KBP: Knowledge-based plan; OAR: Organ-at-risk; PCM: Pharyngeal constrictor muscle; UES: Upper esophageal sphincter

**Figure 5:**
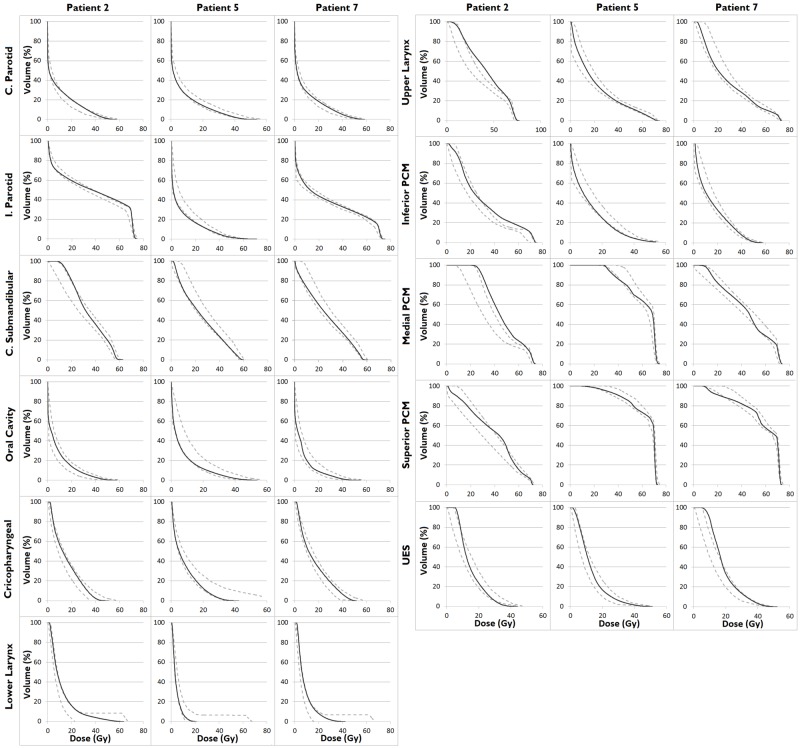
Prediction ranges (grey dashed lines) and DVHs (black lines) for the KBPs of three evaluation patients C. Parotid: Contralateral parotid; I. Parotid: Ipsilateral parotid; C. Submandibular: Contralateral submandibular; DVH: Dose-volume histogram; KBP: Knowledge-based plan; PCM: Pharyngeal constrictor muscle; UES: Upper esophageal sphincter

Model quality, based upon the provided metrics, was good. R^2^ values for OARs were 0.76-0.93, with at least 43 structures matched to their respective OAR model structure (Table 1). Figure 2 shows the regression plots of 10 OARs. Visually, good correlation was seen between both the geometric and dosimetric features of regression plots. Additionally, for 9 OAR regression models the “Geometric distribution principal component score 1” alone (or in combination with another geometric feature) explained most of the variation in the dosimetry - as seen on the horizontal axes of the regression plots. The accuracy of model generated DVH-predictions is illustrated in Figures 3 and 4. Achieved OAR mean dose showed good agreement with predicted mean dose in all KBPs with differences of <3 Gy for 88 of 109 OARs. Of the remaining 21 OARs, six were flagged as geometric outliers. Figure 4 shows that the largest differences between predicted and achieved mean dose occurred predominantly in swallowing OARs. KBP DVHs typically lay within the predictions, which is shown in Figure 5 for three evaluation cases where patients 5 and 7 were arbitrarily selected while patient 2 was selected as it contained the largest difference in predicted-achieved mean dose. This difference occurred in the medial pharyngeal constrictor muscle (PCM), for which the predicted mean dose was 7 Gy lower than the achieved mean dose. This medial PCM had a volume of only 1 cm^3^ compared with the average volume in the model of 3.5 cm^3^.

**Table 1:** R2 values, numbers (#) and volumes of organs-at-risk in the model and the volume of organs-at-risk in the 10-patient evaluation group C. Parotid: Contralateral parotid; I. Parotid: Ipsilateral parotid; C. Sub: Contralateral submandibular; PCM: Pharyngeal constrictor muscle

Model					Evaluation Group
Organ-at-risk	R^2^	#	Volume (cm^3^)		Volume (cm^3^)
C. Parotid	0.77	46	27.6 ± 8.9 (12.3 - 46)		32.2 ± 10.6 (21.2 - 52.4)
I. Parotid	0.78	47	28 ± 8.2 (11.6 - 47.1)		30.7 ± 10.8 (18.9 - 55.5)
C. Sub.	0.9	43	9.3 ± 2.6 (4.1 - 14.9)		9.6 ± 2.4 (5.9 - 13.6)
Oral Cavity	0.76	50	152.8 ± 84.9 (21.1 - 362.3)		103.7 ± 19.4 (57.7 - 128.8)
Cricoph	0.91	50	3.1 ± 1.4 (0.7 - 7.8)		3.4 ± 1.5 (1.3 - 5.8)
Lower Larynx	0.93	49	6.3 ± 5.5 (1.5 - 27.3)		6 ± 3.5 (1.4 - 12.2)
Upper Larynx	0.89	48	11.6 ± 5.5 (4.4 - 30.3)		10.6 ± 8 (2.9 - 29.8)
Inferior PCM	0.92	49	4.2 ± 1.8 (1.4 - 9.7)		3.1 ± 1.4 (0.7 - 5.9)
Medial PCM	0.85	49	3.5 ± 2.6 (0.8 - 12.9)		2.5± 1.2 (0.8 - 4.5)
Superior PCM	0.86	49	7.3 ± 3.5 (0.8 - 17.4)		5.6 ± 0.7 (4.7 - 7)
Upper esophageal sphincter	0.8	47	1.6 ± 0.9 (0.7 - 5.9)		3.1 ± 2.5 (0.7 - 7.8)

DVH predictions required 0.7 minutes while optimization and dose calculation of KBPs required 4.1 minutes, on average (n=5). A “continue optimization” (used to improve PTV dose/homogeneity [12] in 8 of 10 cases) and dose calculation could take another 2.8 minutes (the time necessary to build the model-library is not included in these figures). KBPs were generally of comparable quality to manually created plans. Averaged over all evaluation patients, HI_B_ and HI_E_ differed by <0.6% between manual and KBPs. Only 2 of 109 OARs had a >3 Gy increase in mean dose in the KBPs compared to manual plans: the first was a 4.2 Gy increase to the contralateral submandibular gland of patient 1 (the predicted mean dose was 3.5 Gy higher than the KBP achieved mean dose, it was not flagged as a geometric outlier). At the same time, KBP HI_E_ improved by 1.7% and comp_swal_/oral cavity mean dose decreased by 0.9 Gy/1.2 Gy over that of the manual plan; the second concerned a 4.4 Gy higher mean dose to the lower larynx of patient 10 (this was flagged as geometric outlier on the basis of its small volume).

**Figure 6:**
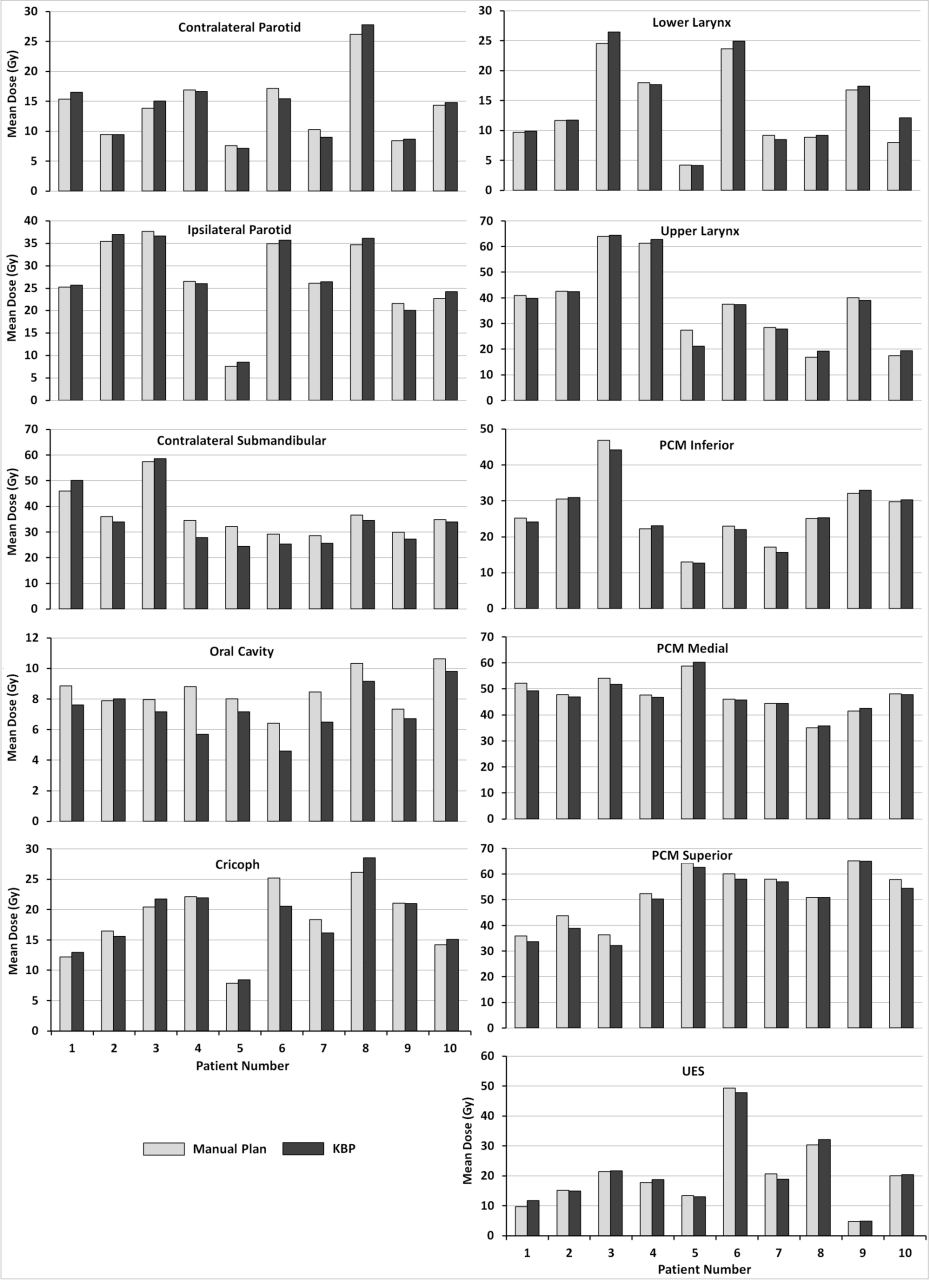
OAR mean doses for the 10 evaluation patients using manual plans (grey) and KBPs (black) KBP: Knowledge-based plan; OAR: Organ-at-risk; PCM: Pharyngeal constrictor muscle; UES: Upper esophageal sphincter

Figure 6 shows the mean dose to OARs for individual patients. There was a statistically significant reduction in the oral cavity and superior PCM mean dose when using KBPs over manual plans, 1.2 Gy and 2.1 Gy on average, respectively. Maximum dose to the shoulders, spinal cord and brainstem differed by <3 Gy, on average, between manual plans and KBPs.

## Discussion

This article described the key features of a prototype proton-specific automated IMPT planning solution and reported the results of an evaluation for HNC. The model/knowledge-based solution showed a relatively high degree of accuracy in predicting the achieved mean OAR dose and plan quality was comparable to that of manually made IMPT plans with, generally, minimal differences in PTV homogeneity and OAR sparing. In addition, the automated plans required, on average, approximately 4-7 minutes for creation (7 minutes includes a “continue previous optimization” function). Similar to the photon solution, this automated IMPT planning solution has numerous potential advantages, including (i) improving planning efficiency, (ii) reducing planning variation [4], (iii) utilization in the quality assurance of proton plans and clinical trial plans [14-15] and (iv) the possibility of sharing planning expertise between proton centers [16]. Knowledge sharing might help to overcome the IMPT learning curve and allow centers to benchmark their own plans [17-18]. This IMPT prototype solution also enables physicians to rapidly, and accurately, estimate the magnitude of possible improvements with proton therapy, over photons, for individual patients [8].

The fact that the geometric principal component score 1 appeared in the majority of regression plots (Figure 1), which showed a high degree of correlation with DVH principal component score 1, suggests that the GED alterations by incorporating the physical characteristics of protons to evaluate the dose to an OAR based on its distance to the target, is functioning well. Although there were a number of deviations between predicted and achieved mean dose for KBPs, these deviations were not always accompanied by a geometric outlier warning (Figure 4), emphasizing that the resulting plans still require additional quality checks. These checks should include target dose homogeneity, which, in certain cases, required improvement through the use of a ‘continue previous optimization’ step.

This study has a number of limitations. All HNC patients, both evaluation cases and those in the model library, had a standardized field set up. Since many proton centers may use differing beam arrangements, to determine the versatility of this planning solution, future work should investigate how the standardized plans compare to alternative beam arrangements and whether implementation of automated beam-angle selection is feasible. Furthermore, in this study, we used the PTV_COMP_ as the field-target for all fields whereas a proton center may split the target into parts and utilize different field-targets per field direction depending on the PTV geometry/location. Additionally, all IMPT plans in this study were non-robustly optimized. While preliminary results on the use of RapidPlan^TM^PT in creating robustly optimized IMPT plans for external proton centers are promising [19], a dedicated investigation should be considered in future to accommodate the increasing interest in robust optimization. Finally, in this investigation, we report on the physical dose. Further development of proton treatment planning applications may enable the potential variation in the relative biological effectiveness (RBE) along the SOBP, especially towards the distal edge, to be accounted for. Recent work suggests that the conventionally used RBE of 1.1 appears valid for the mid-SOBP region but higher values more distally could be clinically relevant [20].

Automated IMPT planning is in its infancy, however, Hall et al. constructed a geometric knowledge-based model to predict patient-specific improvements using protons, over other modalities, for clival chordoma patients. Their model was based upon the correlation between dose and the distance-to-target, and used to predict feasible OAR DVHs for new patients [21]. Hennings et al. developed a tool to automatically pre-calculate feasible planning solutions for uveal melanomas [22]. Meanwhile, Bijman et al. investigated uncertainties in model-based patient selection for IMRT or IMPT, using automatically planned IMPT plans. Their approach to automation differed to that seen in this study, in that it incorporated a pre-defined wish-list of hard constraints and hierarchical OAR objectives (tackled in order of priority) [23].

## Conclusions

This investigation details a pre-clinical knowledge-based IMPT planning solution. This solution demonstrated efficiency and consistency while creating IMPT KBPs which were comparable to manually created treatment plans. Since inferior OAR sparing in a KBP was not always combined with geometric-outlier warnings, manual checking of plans remains important. Such an automated treatment planning solution has several potential applications, including assistance in clinical trial quality assurance and diminishing the learning curve associated with IMPT treatment planning.

## References

[1] Nelms BE, Robinson G, Markham J (2012). Variation in external beam treatment plan quality: an inter-institutional study of planners and planning systems. Pract Radiat Oncol.

[2] Verbakel W, Raaijmakers N, Bos L (2018). Dutch national head and neck plan comparison significantly improved treatment plan quality. Estro35.

[3] Langendijk JA, Lambin P, De Ruysscher D (2013). Selection of patients for radiotherapy with protons aiming at reduction of side effects: the model-based approach. Radiother Oncol.

[4] Tol JP, Delaney AR, Dahele M (2015). Evaluation of a knowledge-based planning solution for head and neck cancer. Int J Radiat Oncol Biol Phys.

[5] Delaney AR, Dahele M, Tol JP (2017). Knowledge-based planning for stereotactic radiotherapy of peripheral early-stage lung cancer. Acta Oncol.

[6] Fogliata A, Wang P, Belosi F (2014). Assessment of a model based optimization engine for volumetric modulated arc therapy for patients with advanced hepatocellular cancer. Radiat Oncol.

[7] Fogliata A, Nicolini G, Bourgier C (2015). Performance of a knowledge-based model for optimization of volumetric modulated arc therapy plans for single and bilateral breast irradiation. PLoS One.

[8] Delaney AR, Dahele M, Tol JP (2017). Using a knowledge-based planning solution to select patients for proton therapy. Radiother Oncol.

[9] Varian Medical Systems (2014). Eclipse Photon and Electron Reference Guide. Eclipse Photon and Electron Reference Guide.

[10] Tol JP, Dahele M, Delaney AR (2016). Detailed evaluation of an automated approach to interactive optimization for volumetric modulated arc therapy plans. Med Phys.

[11] Fogliata A, Belosi F, Clivio A (2014). On the pre-clinical validation of a commercial model-based optimisation engine: application to volumetric modulated arc therapy for patients with lung or prostate cancer. Radiother Oncol.

[12] Tol JP, Dahele M, Doornaert P (2014). Toward optimal organ at risk sparing in complex volumetric modulated arc therapy: an exponential trade-off with target volume dose homogeneity. Med Phys.

[13] Delaney AR, Tol JP, Dahele M (2016). Effect of dosimetric outliers on the performance of a commercial knowledge-based planning solution. Int J Radiat Oncol.

[14] Tol JP, Dahele M, Delaney AR (2015). Can knowledge-based DVH predictions be used for automated, individualized quality assurance of radiotherapy treatment plans?. Radiat Oncol.

[15] Li N, Carmona R, Sirak I (2017). Highly efficient training refinement, and validation of a knowledge-based plan quality control system for radiotherapy clinical trials. Int J Radiat Oncol.

[16] Berry SL, Ma R, Boczkowski A (2016). Evaluating inter-campus plan consistency using a knowledge based planning model. Radiother Oncol.

[17] Mohan R, Das IJ, Ling CC (2018). In reply to Dahele et al. Int J Radiat Oncol Biol Phys.

[18] Liao Z, Lee JJ, Komaki R (2018). Bayesian adaptive randomization trial of passive scattering proton therapy and intensity-modulated photon radiotherapy for locally advanced non-small-cell lung cancer. J Clin Oncol.

[19] Delaney AR, Dong L, Mascia A (2018). Automated knowledge-based intensity-modulated proton planning: an international multicenter benchmarking study. Cancers.

[20] Saager M, Peschke P, Brons S, Debus J, Karger CP (2018). Determination of the proton RBE in the rat spinal cord: Is there an increase towards the end of the spread-out Bragg peak?. Radiother Oncol.

[21] Hall DC, Trofimov A V., Winey BA, Liebsch NJ, Paganetti H (2017). Predicting patient-specific dosimetric benefits of proton therapy for skull-base tumors using a geometric knowledge-based method. Int J Radiat Oncol Biol Phys.

[22] Hennings F, Lomax A, Pica A, Weber DC, Hrbacek J (2018). Automated treatment planning system for uveal melanomas treated with proton therapy: a proof of concept analysis. Int J Radiat Oncol.

[23] Bijman RG, Breedveld S, Arts T (2017). Impact of model and dose uncertainty on model-based selection of oropharyngeal cancer patients for proton therapy. Acta Oncol.

